# Data to estimate costs of producing grass-clover silages

**DOI:** 10.1016/j.dib.2020.106003

**Published:** 2020-07-07

**Authors:** Ola Flaten, Daniel Muluwork Atsbeha, Tor Lunnan

**Affiliations:** Norwegian Institute of Bioeconomy Research, P.O. Box 115, Aas NO-1431, Norway

**Keywords:** Economic analysis, Cost of production, Grassland, Yield, Forage quality, Cutting system, Mountain farming

## Abstract

This article presents input data and procedures used to estimate costs of producing grass-clover silages under Norwegian farming conditions. Data of yield, botanical composition and forage quality of the grass crop were derived from a field experiment comparing a three-cut system, harvested at early crop maturity stages producing highly digestible forages, and a two-cut system returning higher herbage yields of medium digestibility. Secondary data on prices of specific inputs were also provided. The data presented here can be used by advisors and farmers as a decision support tool for assessing and comparing costs of different ways of producing silage. Cost estimates of home-grown forages are also needed in bio-economic evaluations of grassland production and utilization by researchers. The data presented is related to the research article entitled: “Technical and economic performance of alternative feeds in dairy and pig production” [1].

Specifications Table

**Table:** 

**Subject**	Agricultural and Biological Sciences (General)
**Specific subject area**	Production economics, farming system analysis
**Type of data**	Tables
**How data were acquired**	Experimental data from field plots used for estimating herbage yields and composition of the grass-clover silage and supplemented with data from [2] and industry sources. Data were processed and cost budgets of the cropping systems were developed in Microsoft Office Excel.
**Data format**	Raw, filtered and analysed
**Parameters for data collection**	Fields were established in 2003 and 2004, and records were kept for the following four years. Price and cost data were collected from [2] and industry sources.
**Description of data collection**	The experimental plots were harvested two or three times per season. At all harvests, the crop was cut to a stubble height of 5 cm. Herbage yields, botanical composition and feed quality from each cut were recorded. Near-infrared reflectance spectroscopy (NIRS) was used to determine content of neutral detergent fibre (NDF), indigestible NDF, ash, in vitro true digestibility (IVTD) and N-concentration. Secondary data were collected to represent costs of various operations, used to estimate the total cost of producing silage.
**Data source location**	Institution: Norwegian Institute of Bioeconomy Research City/Town/Region: Løken Research Station, Øystre Slidre, Innlandet Country: Norway Latitude and longitude (and GPS coordinates) for collected samples/data: 61°8′N, 9°8′E
**Data accessibility**	With the article
**Related research article**	D.M. Atsbeha, O. Flaten, H.F. Olsen, N.P. Kjos, A. Kidane, A. Skugor, E. Prestløkken, M. Øverland, Technical and economic performance of alternative feeds in dairy and pig production. Livest. Sci. 240 (2020), 104123. https://doi.org/10.1016/j.livsci.2020.104123

## Value of the data

• Data on herbage yields, prices and inputs used in the production of grass are useful to estimate and compare costs of different cutting systems of silage making.
• These data can benefit researchers, advisors, policy makers and farmers who have interest in costs of producing grass silage of different quality with respect to digestibility and protein content.
• For further insights the data may be used in integrated whole-farm system approaches where the most efficient way of using resources in grass production is considered simultaneously with how best to use them in livestock production.

## 1 Data description

The cost of forage varies enormously depending on the growing conditions, soil fertility and type, intensity of farming practice and managerial ability [3]. The primary data presented here were collected from a field experiment at Løken Research Station, located in the mountain region of Eastern Norway, conducted to quantify the relationship between cutting systems and the associated herbage yields, persistence and chemical composition of grasses. The data comprised filtered and analysed raw data. Periodical data recorded from the multi-years field experiment conducted at two different sites are described for the study years.

Table 1 shows the harvesting regimes used in the experiment.

**Table 1:** Harvesting regimes at Løken Research Station.

Regime	1. cut	2. cut	3. cut
1	Onset of stem elongation	500 day-degreesa^a^	August 30
2	One week before early heading	400 day-degrees	August 30
3	One week before early heading	500 day-degrees	August 30
4	One week before early heading	600 day-degrees	August 30
5	Early heading	400 day-degrees	August 30
6	Early heading	500 day-degrees	August 30
7	Early heading	600 day-degrees	August 30
8	Full heading	August 30	–

a Day-degrees were accumulated with base temperature 0 °C.

The grass experiment database is in Microsoft Excel format (file: ‘field_experiment’ in Supplementary material) and it contains seven sheets. The first sheet (‘Fields’) is an overview of the harvesting regimes and distribution of fertilizer N. Herbage yields for the different harvest regimes and N fertilization (120 or 240 kg N per ha per season) by site, year and cut are found in the second sheet (‘DM-yields’). Corresponding quality parameters (content of neutral detergent fibre (NDF), indigestible NDF, dry matter digestibility, crude protein, and ash) and botanical composition are found in sheet 3 (‘Forage_quality’) and sheet 4 (‘Bot_comp’), respectively. The fifth (‘120_N’) and sixth sheet (‘240_N’) shows the average (yield-weighted) yields and quality parameters of the two sites, respectively. Sheet 5 and 6 are limited to the harvesting regimes (4,7, and 8) used directly or indirectly in the calculations of production costs. The seventh sheet (‘Summary_farm_yields’) shows the calculations of the herbage yields adjusted to farm-level conditions.

Table 2 presents data on government farm payments and prices on inputs needed to produce grass-clover silage. Based on the parameters, unit costs of the two grass silage types have been calculated. Yield and costs of production are presented in Table 3. The system for comparing costs between different cutting regimes for silage making are found in a Microsoft Excel sheet (file: ‘Cost_silage’ in Supplementary material). The first two sheets (‘Inputprice’ and ‘Machinery’) contain the input prices. Sheet 3 (‘Sward_est’) quantifies the per hectare costs in the establishment year and sheet 4 (‘Ley_years’) calculates the per-hectare costs in the later sward years (average of the years). Total costs of the preserved silages are calculated in sheet 5 (‘Total_cost’). The steps involved for the calculation of the costs are described in Section 2.3.

**Table 2:** Government farm payments and input prices to produce grass-clover silage.

Item	Value (NOK)	Item	Value (NOK)
*Governmental payments*		Spraying	250/ha
Grassland	4010/ha	Mowing	450/ha
*Expenses*		Raking	250/ha
Land rent	2500/ha	Custom baling^a^	180/bale
*Machinery, contract charges*		*Other expenses*	
Ploughing	1000/ha	Seed	52/kg
Harrowing	300/ha	Herbicide (MCPA)	58/ha
Rolling	250/ha	Fertilizer (NPK 18–3–15)	4.14/kg
Dragging	450/ha	Lime^b^	0.65/kg
Seeding	450/ha	Silage additive	10/l
Fertilizer spreading	200/ha		

a Wrapping and transport of bales included.

b Cost of lime includes material, hauling it to the field and application. Limestone is applied at an average annual rate of 690 kg per ha. Source: NILF [2]. Exchange rates in 2014 was NOK 100 = $ 15.87 = € 11.97 = £ 9.64.

**Table 3:** Yields, composition of silage and total costs (NOK/kg dry matter, DM) of wrapped grass silage.

Cost of preserved silage	2-cut	3-cut
Yield and composition of silage		
Yield, farm-level (kg DM/ha)	6243	4785
Crude protein (g/kg DM)	105	143
Neutral detergent fibre (g/kg DM)	581	520
Dry matter digestibility (% of DM)	67.1	72.8
Costs (NOK/ha)		
Seed	260	325
Fertiliser	3765	3647
Sprays	12	14
Mowing	810	1125
Raking	450	625
Drilling	90	113
Land preparation	490	613
Fertilising and lime	797	934
Spraying	50	63
Land rent	2500	2500
Area payment	−4010	−4010
Land based costs (NOK/ha)	5213	5948
Sub-total (NOK/kg DM)	0.84	1.24
Baling (NOK/kg DM)	0.72	0.72
Preservatives (NOK/kg DM)	0.10	0.10
Total costs (NOK/kg DM)	1.66	2.06
Total costs, adjusted for losses^a^	1.84	2.29

a A 10% loss of DM and nutrients from silages during storage and feed-out was assumed (cf. Flaten et al. [4]). *Notes*: Costs of seed, sprays, land preparation, drilling and spraying are divided by length of rotation (5 years for 2-cut and 4 years for 3-cut). Other land-based costs and yield are weighted by the establishment year and later years in the rotation. Swards are mown, raked and fertilized three times in the 3-cut system compared to twice for the 2-cut system.

## 2 Experimental design, materials, and methods

### 2.1 Design of field experiment and establishment of the grass-clover crop

The field experiment was conducted at two different sites at Løken Research Station (61°8′N, 9°8′E, altitude 530 (Fjøsjordet) and 450 m (Eikra) above sea level, 590 mm precipitation, 149 growing days, 1961–1990 averages) in the mountain region of Eastern Norway.

The crop was sown with an experimental row drill at a seeding rate of 25 kg per ha, one in June 2003 (Site 1 Fjøsjordet) and one in June 2004 (Site 2 Eikra). The seed mixture contained (w/w) 40% timothy (*Phleum pratense*, cvs Grindstad and Vega), 40% meadow fescue (*Festuca pratensis Huds.*, cv. Fure), and 20% red clover (*Trifolium pratense* L., cv. Nordi). In the establishment year, yields were not measured. Records were kept for the following four years with 2008 as the last year.

Six or eight different harvesting regimes were used (Table 1). Five or seven regimes were with three cuts and one with two cuts. Regime 3 and 6 with 500 day-degrees before the second cut were only tested in Site 2. The first cuts were determined by the phenological stage of timothy, and the following second and third cuts was determined after accumulated heat sum (day-degrees) or date. The phenological stage of development at harvesting was expressed as mean stage by count (MSC; [5]). The swards were not grazed late in season.

The harvesting regimes were combined with two nitrogen application levels, N1 = 120 kg N per hectare and N2 = 240 kg N per hectare in each of the established sward years. Fertilizers were distributed between spring and regrowths according to expected share of total herbage yield. Nutrients were applied as inorganic NPK 18‐3‐15 (NPK) compound fertilizer (N in the form of both ammonium and nitrate; [6]). At both sites, the experiment was laid out in a completely randomized block design with 6–8 harvesting regimes × two fertilizer rates. There were three replicates.

At all harvests, the crop was harvested with an Agria two-wheel tractor (Agria‐Werke GmbH, Mockmuhl, Germany) with the blade set to a stubble height of 5 cm.

### 2.2 Records

Yield was determined based on raw weight. The dry matter (DM) content of the yield was determined by drying a subsample of approximately 1 kg at 60 °C for 48 h. Dry-matter yields across harvest were summed within each year to give the annual DM yield per plot.

Botanical composition was evaluated visually in all individual plots, and in addition with sorting of samples taken from selected plots. There was no sorting in 2007.

The nutritive value of the harvested herbages was measured for each cut. Most often, samples were analysed from two replicates. The grass samples were ground to 1 mm (Cyclotec™ Sample Mill 293) and then scanned using a near-infrared spectrophometer (NIRS; [7]) (NIR systems 6500, Silver Spring MD, USA) at Løken Research Station to determine neutral detergent fibre (NDF), indigestible NDF, in vitro organic DM digestibility, ash and N-concentration. Crude protein (CP) concentration was calculated by multiplying the N concentration by 6.25.

More details on the design of the field experiment, herbage yield and feed quality parameters are described in [8].

### 2.3 Costing

The swards were established in the spring after ploughing and conventional cultivation for seedbed preparation without a cover crop. Establishment year field operations included (number of operations in parenthesis): ploughing (1), dragging (2), harrowing (1), drilling (1), rolling (1), spraying (1), fertilizing (1), mowing (1), raking (1), baling (1), and hauling forage (1). One herbicide treatment with MCPA was needed to control annual weeds and 80 kg N per ha (using NPK 18 – 3- 15) was applied.

The establishment year fields were harvested for silage making, but no yield or forage quality records were kept. Expert guestimates were used to assess herbage yields in the establishment year. Yields were assumed to be 50% of the annual yields in the established sward years of the three-cut and two-cut system (with 120 kg N/ha), respectively. Herbage harvested in the establishment year was assumed to be of similar feed quality to that of the later sward years in the respective cutting system. The assessed herbage yields in the year of sward establishments are reported in the Supplementary material (file: ‘field_experiment’; sheet: ‘Summary_farm_yields’).

Substantial stand and yield losses occurred in the last years of the three-cut systems, irrespective of fertiliser input, and weeds, especially *Taraxacum officinale*, invaded these plots. For the costing of the three-cut system we used the average yields from the first three sward years of the experiment and in consequence we assumed ley duration of three years (the establishment year excluded). For the two-cut system we used four-year yield averages and ley duration of four years, that is, a total life span of five years. Swards used for two-cut and three-cut silage systems were thus reseeded every 5 and 4 years, respectively.

In the cost analysis, the established swards annually received 180 kg N per ha (average of 120 kg N per ha and 240 kg N per ha used in the field experiment). Plant nutrients came from bagged fertilizer. The number of field operations with fertilizing, mowing, raking in the established sward years were equal to the number of cuts of the harvesting regime.

Forage quality parameters of both types of silage used in the dairy cow experiment described in [1] were close to the phenological stages, digestibility and the CP content of the yields of the two-cut and one of the three-cut systems at Løken Research Station used in Flaten et al. [4]. Therefore, we considered it to be appropriate to use these two- and three-cut systems too estimate the costs of feeding dairy cows with low crude protein silage and optimal crude protein silage in study [1], respectively. The two-cut system in [4] were represented by harvesting regime 8 (Table 1), whereas the three-cut system was composed of a 50:50 mixture of harvesting regime 4 and harvesting regime 7.

In general, it is well known that responses under experimental conditions significantly exceed the responses achieved under commercial farm conditions [9]. As in Flaten et al. [4], we adjusted farm DM yields to 60% of the experimental yields. The resulting farm-level DM yields and energy and protein concentration of the grasses harvested are shown in Table 3.

The grass was conditioned at mowing, wilted and raked before harvesting. DM content of the wilted silage was 40%. The silage was preserved with the silage additive GrasAAT Plus (580 g formic acid, 12 g propionic acid, and 1.5 g benzoic acid per kg; Addcon Nordic AS, Porsgrunn, Norway) applied at 4 l per t fresh weight of wilted crop prior to ensiling, and it was wrapped into round bales (625 kg per bale) using 8 layers of stretch-film.

Costs for all field operations were taken from contractor's charges. The prices of all inputs to produce the grass silage, which are reproduced in Table 2, were set to reflect 2014-conditions.

The costing included a land charge for the land which was required to produce the silage. We assumed that the farmer had access to enough own or rented land. With more restrictive availability of land, forage will become a scarcer resource. The approach therefore may ignore the opportunity cost of land needed to produce feeds on a farm. Farmers are also paid a premium per ha of farmland, which are deducted from the cost of producing silage.

The land-based costs (per hectare) were calculated first. In determining land-based costs, cost only occurring in the establishment year (seed, sprays, drilling, land preparation, and spraying) were divided by length of rotation. Other land-based costs (fertilizer, lime and fertilizing, mowing, raking, and land rent) and area payments were weighted by the establishment year and later years in the rotation. Summing these items gave the total land-based costs per hectare (Table 3). Next, the land-based costs were converted to cost per kg DM yield. Costs associated with baling and hauling of the harvested forage and the use of silage additives are constant per unit DM produced, and these cost items were added directly to the per-unit total costs. Finally, the costs were adjusted for losses incurred during fermentation and storage and during feeding-out.

There may be significant cost savings for livestock farmers from feeding concentrates as compared to grass silage in terms of labour. Feed-out labour considerations are difficult to quantify and varies a lot between farms and feed-out systems, and feed-out labour costs of feeds were not considered here.

Based on these calculations, the total cost of producing silage were NOK 1.84 per kg DM for the two-cut system and NOK 2.29 per kg DM for the three-cut system (Table 3), and in [1] these estimates are used to represent the cost of low crude protein silage and optimal crude protein silage, respectively.

## Declaration of Competing Interest

The authors declare that they have no known competing financial interests or personal relationships which have, or could be perceived to have, influenced the work reported in this article.
